# Oral Manifestations of Prediabetes: A Narrative Review of an Early Diagnostic Window for Dental Practice

**DOI:** 10.7759/cureus.109375

**Published:** 2026-05-21

**Authors:** B Roshan Arbaaz, Thangadurai Maheswaran, Subramanian Adalarasan, Thangavelu Kavin, Kaliyamurthy Sivaguru, Georgebabu Jisha

**Affiliations:** 1 Periodontics, Sri Venkateshwaraa Dental College, Puducherry, IND; 2 Oral and Maxillofacial Pathology, Adhiparasakthi Dental College and Hospital, Melmaruvathur, IND; 3 Oral and Maxillofacial Surgery, Government Dental College and Hospital, Chidambaram, IND; 4 Oral and Maxillofacial Surgery, Vivekanandha Dental College for Women, Namakkal, IND; 5 Prosthodontics, No. 1 Dental Unit, Assam Rifles, Shillong, IND; 6 Oral and Maxillofacial Pathology, Nandha Dental College and Hospital, Erode, IND

**Keywords:** clinical attachment loss, diabetes screening, glycemic control, metabolic dysfunction, oral manifestations, oral microbiome, periodontitis, prediabetes, salivary biomarkers

## Abstract

Prediabetes is a common metabolic condition affecting a substantial proportion of adults worldwide; however, most individuals remain undiagnosed owing to the prolonged asymptomatic phase of this condition. The oral cavity generates a constellation of clinically detectable changes during this intermediate glycemic stage, including periodontal attachment loss, salivary dysfunction, and oral microbiome dysbiosis, all of which can be examined by dental professionals. Periodontal clinical attachment loss and elevated glycated hemoglobin (HbA1c) levels show a dose-dependent relationship in nondiabetic populations, and active matrix metalloproteinase-8 (aMMP-8) in oral fluids correlates independently with prediabetes, even in its early stages. Salivary oxidative stress markers, adipokines, cariogenic bacterial loads, and physicochemical parameters are measurably altered before overt hyperglycemia is established. Oral microbial communities show reduced species richness, altered IgA immune responses, and shifts in keystone taxa in prediabetic individuals compared to those in healthy individuals. Chairside HbA1c measurement, aMMP-8 point-of-care testing, and structured interprofessional referral models have demonstrated high diagnostic yields in real-world dental settings. This narrative review explores the current literature and proposes dental practice as a first-line venue for opportunistic prediabetes detection, with an emphasis on actionable clinical strategies for practicing dentists.

## Introduction and background

The global burden of dysglycemia is increasing at an alarming rate worldwide. In 2021, the International Diabetes Federation (IDF) estimated that 537 million adults were living with diabetes, with tens of millions more in the prediabetic range of glucose dysregulation, which often goes undetected for years [1-3]. Prediabetes is characterized by a fasting plasma glucose (FPG) level of 100-125 mg/dL, a two-hour oral glucose tolerance test result of 140-199 mg/dL, or a glycated hemoglobin (HbA1c) level between 5.7% and 6.4%. Despite its reversibility, it is associated with the early onset of microvascular complications, cardiovascular risk, and progressive β-cell failure [3,4]. The asymptomatic nature of prediabetes and the documented diagnostic delay of two to seven years between metabolic onset and clinical recognition mean that a large proportion of affected individuals remain invisible to the healthcare system [2,5]. This diagnostic gap carries serious consequences: up to half of people with diabetes and the vast majority of those with prediabetes are unaware of their condition, and vascular end organ injury may occur long before any formal diagnosis is made [2,5].

The oral cavity mirrors systemic metabolic health in multiple dimensions that are directly relevant to the prediabetic state [5,6]. Chronic subclinical hyperglycemia promotes an exaggerated host inflammatory response to periodontal pathogens, accelerated accumulation of advanced glycation end products (AGEs), impaired salivary gland secretory function, and measurable shifts in the oral microbial ecosystem [1,7,8]. These mechanisms lead to clinically noticeable oral symptoms, such as loss of periodontal attachment, dry mouth, fissured tongue, burning sensation in the mouth, and increased levels of chairside active matrix metalloproteinase-8 (aMMP-8). These symptoms may appear before or at the same time as the metabolic threshold that marks the onset of overt prediabetes [5,9,10]. The detection of these signs during routine dental examinations creates a clinically meaningful and low-cost opportunity to identify the glycemic risk before the development of irreversible complications.

Dental practice is a structurally underutilized venue for the early identification of metabolic risk [2,11]. In many high-income countries, a larger proportion of adults visit a dental professional at least once annually than visit their primary care physician, and chairside HbA1c devices require only a finger-prick capillary blood sample with a turnaround time of five minutes or less [2,11]. Interprofessional screening models linking dental and primary care services have demonstrated high diagnostic yields in real-world deployments across multiple healthcare systems [3,12]. Despite this convergence of clinical evidence, systematic glycemic screening remains uncommon in dental practice, partly because of regulatory heterogeneity, the absence of reimbursement frameworks, and limited awareness among practitioners of the strength of the oral-metabolic link [13].

This narrative review examines the current evidence on the principal oral manifestations of prediabetes and evaluates the established and emerging strategies for the identification of prediabetes in the dental practice setting.

## Review

Prediabetes: definition, epidemiology, and the undiagnosed burden

Prediabetes represents a reversible metabolic state in which blood glucose is elevated beyond the normal limits but has not yet reached the diagnostic threshold for type 2 diabetes mellitus (T2DM) [3,4]. The American Diabetes Association (ADA) defines (in 2010) prediabetes as an FPG level of 100-125 mg/dL, a two-hour post-load glucose level of 140-199 mg/dL, or an HbA1c level between 5.7% and 6.4%, criteria that the IDF and World Health Organization apply with minor regional variations. Despite this reversibility, complications of T2DM, including retinopathy, peripheral neuropathy, and chronic kidney disease, may begin to develop in the prediabetic stage, underscoring the clinical urgency of early detection [1,3].

Globally, the IDF estimates that half of all diabetes cases remain undiagnosed, and the number of unrecognized prediabetes cases is much larger [1,2]. In Sweden, approximately one in three diabetes cases is undetected, and the total diabetes prevalence is 5-6% of the population [3]. A meta-analysis conducted specifically in the dental setting estimated that 47.38% of screen-positive dental patients had prediabetes confirmed by a reference standard diagnostic test, which is markedly higher than the estimated 4.9% pooled national prevalence of undiagnosed diabetes in the countries represented [2]. The Swedish DentDi study found that 27% of 372 dental patients who completed screening had elevated blood glucose levels, with 89 individuals receiving a new diagnosis of prediabetes [3]. In Pakistan, approximately two-thirds of prediabetic individuals progress to overt diabetes within three years, with a reported diagnostic delay of two to seven years from metabolic onset to recognition [5]. In Malaysia, a structured dental screening program using the Finnish Diabetes Risk Score and capillary fasting blood glucose identified prediabetes in 30.3% of patients who completed the referral pathway from dental to primary care [12]. These figures collectively confirm that the dental patient population is disproportionately enriched with undetected prediabetes compared with the general population.

Periodontal disease as an oral marker of prediabetes

Periodontitis and prediabetes sustain a bidirectional inflammatory relationship, which has been characterized in cross-sectional, longitudinal, and interventional studies [4,6,7]. Chronic hyperglycemia, even at prediabetic levels, drives AGE accumulation in periodontal tissues. The interaction of AGEs with their receptor for AGEs, primarily expressed on macrophages, activates the local immune-inflammatory response and upregulates the secretion of pro-inflammatory cytokines, including IL-1β, tumor necrosis factor-α, and IL-6 [1]. These mediators disrupt the receptor activator of nuclear factor kappa-B ligand/osteoprotegerin axis, favor alveolar bone resorption, and amplify connective tissue destruction, clinically manifesting as increased probing depths, clinical attachment loss (CAL), and bleeding on probing [1,7].

Cross-sectional data from the Oral Infections, Glucose Intolerance, and Insulin Resistance Study (ORIGINS), which enrolled 1,071 nondiabetic younger adults, established that interproximal CAL was independently associated with prediabetes and elevated fasting glucose levels [7]. Compared with the lowest tertile of interproximal CAL, the highest tertile was associated with a prediabetes prevalence ratio of 2.42 (95% CI: 1.77-3.08), with a stepwise increase in fasting glucose across the tertiles (83.29, 84.31, and 86.48 mg/dL, p for trend <0.01) [7]. Separately, interproximal probing depth was associated with insulin resistance as quantified by the Homeostatic Model Assessment for Insulin Resistance (HOMA-IR), indicating that different dimensions of periodontal disease capture distinct facets of the prediabetic metabolic phenotype [7].

In Saudi Arabia, a logistic regression analysis of dental hospital patients demonstrated that each percentage-unit increase in sites with CAL >3 mm raised the likelihood of prediabetes or T2DM by a factor of 1.104, while a positive family history of diabetes increased the odds by 4.98 times, together explaining 47.7% of the variation in glycemic status [6]. In India, a controlled study of nondiabetic individuals with a family history of T2DM found that HbA1c, fasting blood sugar, and C-reactive protein levels were all significantly higher in patients with periodontitis than in periodontally healthy controls, and all three measures declined meaningfully three months after nonsurgical periodontal therapy (NSPT), consistent with the hypothesis that periodontal inflammation actively elevates glycemic indices [14].

The 2017 World Workshop on the Classification of Periodontal and Peri-Implant Diseases and Conditions provided a clinically actionable gradient for periodontitis staging. In a cross-sectional study of 135 nondiabetic adults, the mean HbA1c was 5.52% in periodontally healthy patients, 6.17% in stage I/II periodontitis, and 6.52% in stage III/IV periodontitis (p < 0.01), with the majority of patients in the periodontitis groups falling within the prediabetes HbA1c range [4]. Fully adjusted linear regression confirmed that stage III/IV periodontitis was associated with a 0.85% absolute increase in HbA1c over periodontally healthy individuals, providing a direct clinical signal for glycemic risk stratification [4]. A clinical risk model for undiagnosed diabetes in an Asian dental population further identified advanced periodontitis (stage III/IV) as one of four independent predictors, alongside BMI, family history of diabetes, and smoking, yielding an area under the curve of 0.717 [15].

The biomarker aMMP-8 provides a biochemical link between periodontal inflammation and glycemic dysregulation. Among Greek adults with stage III grade C periodontitis, aMMP-8 levels correlated positively with prediabetes (Spearman ρ = 0.646, p = 0.044), and prediabetes upregulated MMP-8 expression in severe periodontitis, with latent species further activated into aMMP-8 in a tissue-destructive cascade detectable within five minutes by a chairside point-of-care (PoC) test [9]. In a Nigerian cohort, NSPT significantly reduced aMMP-8 levels in both the prediabetes and diabetes groups (p < 0.001), with stage III and IV periodontitis showing the most pronounced treatment effects on both aMMP-8 and HbA1c levels [1]. These findings reinforce the bidirectional nature of the periodontitis-prediabetes relationship and support the integration of aMMP-8 monitoring into the periodontal metabolic screening workflow.

Table 1 provides a concise summary of oral manifestations in prediabetes.

**Table 1:** Common oral manifestations of prediabetes compared with normoglycemia Summary of oral clinical findings and biomarkers associated with prediabetes, including their relationship with glycemic parameters. aMMP-8, active matrix metalloproteinase-8; CAL, clinical attachment loss; DM, diabetes mellitus; HbA1c, glycated hemoglobin Data derived from references [4-6,9]

Oral manifestation	Findings in prediabetes
Gingivitis	Present in 88.2% of prediabetic, undiagnosed DM patients
Localized periodontitis	80.9% prevalence; HbA1c 6.17% (stage I/II) vs 5.52% (healthy)
Halitosis	88.2% prevalence; positively associated with elevated HbA1c
Xerostomia	16.2% of prediabetics; thick, ropy saliva a common feature
Fissured tongue	7.4% of prediabetics (0% in normoglycemic controls)
Elevated aMMP-8	Positively correlated with prediabetes in stage III grade C (ρ = 0.646, p = 0.044)
CAL >3 mm	Each 1-unit increase raises prediabetes/DM likelihood by a factor of 1.104

Salivary changes in prediabetes

Saliva is an accessible, noninvasive biological fluid whose physicochemical properties and molecular composition reflect the host's systemic metabolic environment, with multiple parameters measurably altered even in the prediabetic stage [10,16]. Oxidative stress is an early and prominent feature of dysglycemia. A cross-sectional study comparing 30 prediabetic, 31 T2DM, and 39 normoglycemic individuals found that salivary malondialdehyde (MDA), a marker of lipid peroxidation, and superoxide dismutase activity were significantly correlated with fasting blood sugar and HbA1c levels in both the prediabetes and T2DM groups [10]. Salivary MDA was an independent determinant of T2DM status relative to healthy controls, with a receiver operating characteristic (ROC) cutoff of 2.8 ng/mL producing a sensitivity of 64.2% and a specificity of 82.1% for T2DM, while intermediate MDA elevations were observed in the prediabetes group [10]. Total antioxidant capacity also correlated significantly with fasting blood sugar (p = 0.02, r = 0.23), suggesting that the balance between oxidative burden and antioxidant defense is progressively disrupted along the normoglycemia-to-diabetes continuum [10].

Adipokines are another class of salivary biomarkers with diagnostic potential for early dysglycemia. A study of 35 prediabetic, 35 diabetic, and 34 normoglycemic participants found significant inter-group differences in salivary leptin, chemerin, and resistin (p < 0.01), while salivary IL-6 levels did not differ significantly [16]. Notably, the prediabetic group exhibited the highest appetite trait scores across the three groups, and salivary chemerin correlated significantly with appetite, irrespective of glycemic status (p = 0.009), suggesting that salivary adipokine profiles reflect early metabolic dysregulation before overt hyperglycemia is established [16].

Salivary flow rate and its physicochemical properties are sensitive indices of glycemic status. Experimental data in streptozotocin-induced diabetic rats demonstrated a significant reduction in salivary flow rate following diabetes induction, with vitamin E supplementation most effectively restoring glandular secretory function (1.0 mL at two weeks vs. 0.3 mL in saline controls), providing mechanistic evidence that oxidative injury underlies hyposalivation in the diabetic milieu [17]. In human subjects, salivary calcium, pH, and unstimulated salivary volume have been documented to decrease with increasing glycemic levels [18]. In prediabetic hypertensive women, salivary calcium correlated significantly with systolic blood pressure (p < 0.01), and this relationship was not observed in normoglycemic subjects, suggesting that salivary composition reflects the combined cardiometabolic risk in this intermediate stage [18]. Clinically, these changes manifest as xerostomia, thick ropy saliva, and burning mouth sensation, all of which are directly observable during routine dental examinations [5].

Downstream salivary consequences include a cariogenic microenvironment. In a study comparing normal, prediabetic, and diabetic groups, *Streptococcus *and *Lactobacillus *colony-forming unit counts were significantly different across all three groups (p < 0.01 for *Streptococcus*, p < 0.005 for *Lactobacillus*), and diabetic subjects showed higher dental caries incidence and lower salivary pH relative to both prediabetic and normoglycemic individuals, with prediabetics occupying an intermediate position [19]. This gradient of deteriorating salivary protective capacity, beginning in prediabetes and accelerating in overt diabetes, supports the view that salivary cariogenic parameters serve as surrogate indicators of progressive glycemic dysregulation [19].

Table 2 provides a concise summary of salivary biomarkers in prediabetes.

**Table 2:** Salivary biomarkers and physicochemical parameters altered in prediabetes Overview of salivary biomarkers and physicochemical changes in prediabetes reflecting oxidative stress, inflammation, and early metabolic imbalance. aMMP-8, active matrix metalloproteinase-8; FBS, fasting blood sugar; HbA1c, glycated hemoglobin; MDA, malondialdehyde; NSPT, nonsurgical periodontal therapy; T2DM, type 2 diabetes mellitus Data derived from references [1,9,10,16-19]

Biomarker/parameter	Direction of change	Clinical significance
MDA	Intermediate increase	Reflects oxidative stress; independent predictor of T2DM; correlates with FBS and HbA1c
Salivary adipokines (leptin, chemerin, and resistin)	Altered; highest appetite in prediabetes	Reflects early metabolic dysregulation before overt hyperglycemia
aMMP-8	Elevated; reduced after NSPT	Dual biomarker of periodontal tissue destruction and glycemic risk
Unstimulated salivary flow rate	Reduced	Contributes to xerostomia and caries susceptibility
Salivary pH and calcium	Decreased	Promotes cariogenic and demineralizing environment
Cariogenic bacteria (*Streptococcus*, *Lactobacillus*)	Intermediate increase	Associated with progressively higher dental caries incidence

Oral microbiome alterations in prediabetes

The oral microbiome constitutes a dynamic ecosystem of approximately 700 microbial species, whose composition is sensitive to the host’s glycemic environment, and dysbiosis may both reflect and contribute to systemic metabolic dysfunction [8,20]. Even before the clinical onset of T2DM, prediabetes is associated with measurable shifts in salivary and supragingival microbial communities that are detectable using sequencing-based methods [20,21].

A Thai study employing 16S rRNA V3-V4 amplicon sequencing identified differential bacterial abundance between normoglycemic and potential prediabetes groups, with specific taxa differing depending on whether HbA1c or FPG criteria were used for classification [8]. Regardless of the criterion, *Streptococcus *was consistently dominant in the normoglycemic groups, suggesting a potential protective or neutral role in healthy glucose homeostasis, which is diminished in the prediabetic oral environment [8]. A Japanese case-matched study of 101 prediabetic and 101 normoglycemic individuals demonstrated significantly lower bacterial species richness in the prediabetes group (observed operational taxonomic unit index, p = 0.042) and a distinct overall salivary microbiota structure (unweighted UniFrac distances, p = 0.009) [20]. Salivary IgA responses against several bacterial genera differed between the two groups: prediabetic individuals showed higher IgA reactivity against certain genera and lower reactivity against others, indicating altered host immune recognition of commensal bacteria at the earliest detectable stage of glucose dysregulation [20].

A comprehensive multi-omics study of supragingival dental plaque from Wisconsin patients, combining 16S rDNA sequencing, metabolomics, lipidomics, and proteomics, identified a higher abundance of *Fusobacterium *and *Tannerella *in individuals with prediabetes and T2DM than in metabolically healthy controls [22]. Cross-omic correlation analysis revealed a strong association between *Lautropia* and phospholipid monomethylphosphatidylethanolamine. In vitro confirmation that this unusual phospholipid is synthesized by *Lautropia mirabilis *illuminates a novel microbial metabolic pathway activated under dysglycemic conditions [22]. In a large cohort of 2,974 Qatari participants spanning nondiabetic, prediabetic, and diabetic categories, salivary microbiota from prediabetic and diabetic individuals showed significant increases in *Campylobacter*, *Dorea*, and *Bacteroidales *relative to nondiabetic controls, with metabolic pathway prediction identifying an overrepresentation of genes associated with fatty acid and lipid biosynthesis in the diabetic group [23].

The enterosalivary nitrate-nitrite-nitric oxide pathway is a well-characterized mechanistic link between oral microbial communities and cardiometabolic risk. The ORIGINS study, which sequenced subgingival dental plaque from 281 diabetes-free adults, found that a one-standard deviation increase in the nitrate-reducing taxa summary score was associated with a 0.09-unit reduction in log-transformed HOMA-IR and a 1.03 mg/dL decrease in FPG levels after full multivariable adjustment [24]. Therefore, these nitrate-reducing bacteria may exert a glycemia-buffering effect through nitric oxide-mediated improvements in insulin sensitivity, a pathway that is progressively disrupted as prediabetes progresses [24]. In obese hyperglycemic individuals, a higher *Firmicutes*/*Bacteroidetes *ratio, a recognized obesogenic oral microbiome trait, was documented relative to lean controls, linking dysbiotic signatures of metabolic diseases across multiple host phenotypes [25].

A longitudinal profiling study tracking the microbiome at four body sites over six years confirmed that insulin-resistant individuals show altered microbial stability and disrupted associations among the microbiome, molecular markers, and clinical features, consistent with the hypothesis that oral dysbiosis precedes and potentiates metabolic disease [21]. Integrated multi-omics analysis in a Chinese prediabetes cohort, examining metagenomics, tongue coating 16S rRNA, and plasma metabolomics, identified 509 intestinal bacterial species and 1,122 bacterial genera in tongue coating samples, with *Flavonifractor plautii* playing a key role in modulating blood glucose through carbohydrate metabolism, highlighting the potential of multi-site oral microbiome sampling in prediabetes risk stratification [26]. T2DM patients with periodontitis demonstrate distinct phyla-level shifts, lower *Firmicutes *and *Actinobacteria *with higher *Fusobacteria *and *Spirochetes*, alongside elevated salivary MMP-8, IL-1β, and resistin, with specific taxa correlating directly with clinical periodontal disease features and host inflammatory analytes [27,28]. These microbial patterns, clearly emerging in T2DM, show analogous, albeit attenuated, trends in prediabetes, underscoring the progressive nature of oral dysbiosis across the glycemic continuum [27].

Dental office screening strategies for prediabetes

Dental practice offers structural advantages for opportunistic prediabetes screening that no other healthcare setting matches in combination: high patient visit frequency, routine performance of periodontal examination, and accessibility of chairside PoC technologies [2,11]. More than 90% of patients with periodontal disease meet the ADA criteria for diabetes screening, and most of them have seen a dentist in the preceding two years, making dental professionals the primary interface with an otherwise clinically invisible high-risk group [2].

Chairside HbA1c measurement is the most extensively studied screening modality in dental settings. A systematic review and meta-analysis estimated that 40.10% of dental patients screened using HbA1c had elevated values, with 47.38% of screen-positive patients subsequently confirmed as prediabetic using a reference standard [2]. A feasibility study conducted at a safety-net dental clinic in New York identified undiagnosed prediabetes in 34.2% and undiagnosed diabetes in 6.6% of high-risk adults screened with a fingerstick HbA1c device, with 64.6% of those with dysglycemia consulting their primary care provider within six months of testing [29]. A cross-sectional analysis of 13,519 patients at a university dental clinic found that 35% met the ADA Diabetes Risk Test criteria for T2DM risk, and among those who underwent PoC HbA1c testing, 34.9% had prediabetes and 5.3% had diabetes [30].

The aMMP-8 PoC mouthrinse test adds diagnostic value when combined with HbA1c-based screening. In a study of periodontal clinic patients fulfilling Centers for Disease Control and Prevention screening criteria, adding the aMMP-8 immunotest to a logistic regression model containing BMI and age raised the area under the ROC curve from 0.683 to 0.759 for prediabetes detection [11]. This rapid, non-invasive, five-minute chairside test simultaneously provides information about periodontal disease severity and glycemic risk and has the further advantage that NSPT demonstrably reduces aMMP-8 levels in prediabetic patients, creating a treatment-monitoring role in addition to its diagnostic application [9,11].

Structured interprofessional referral models have demonstrated their feasibility in diverse healthcare settings. The Swedish DentDi method, which systematically refers dental patients with significant caries or periodontal risk findings to primary health care for HbA1c and FPG testing, identified elevated blood glucose levels in 27% of 372 participants who completed screening, operating entirely within existing clinical workflows without requiring new diagnostic equipment at the dental site [3]. In Malaysia, combining the Finnish Diabetes Risk Score (FINDRISC) questionnaire with capillary fasting blood glucose measurements at private dental clinics identified 36.4% of periodontitis patients as high-risk; of those completing the full referral chain, 51.5% received a new diagnosis of prediabetes or T2DM, although referral compliance was lower in patients from lower socioeconomic backgrounds [12].

Regulatory and reimbursement barriers remain the principal structural impediments to its widespread implementation. A US national survey of state dental directors found that only 46% of responding states considered chairside HbA1c testing within the dental scope of practice, 40.5% expressed uncertainty, and minimal reimbursement existed in most jurisdictions, indicating that evidence-based practice and policy alignment remain substantially out of step [13]. Achieving broader adoption will require coordinated advocacy among dental professional bodies, policymakers, and payers for scope-of-practice clarification, reimbursement inclusion, and undergraduate training in the competencies of glycemic screening.

Table 3 provides a concise summary of screening strategies for prediabetes in the dental setting.

**Table 3:** Dental office screening strategies for prediabetes and undiagnosed diabetes Summary of screening approaches used in dental settings for identifying prediabetes and undiagnosed diabetes, including study design and diagnostic outcomes. ADA, American Diabetes Association; AUC, area under the curve; FBG, fasting blood glucose; FINDRISC, Finnish Diabetes Risk Score; HbA1c, glycated hemoglobin; PCP, primary care provider; PoC, point-of-care; T2DM, type 2 diabetes mellitus Data derived from references [2,3,11,12,15,29,30]

Screening method	Setting/design	Key diagnostic yield
Chairside HbA1c (finger-prick)	Meta-analysis (nine studies)	40.10% hyperglycemia by HbA1c; 47.38% prediabetes confirmed
Chairside HbA1c at safety net dental clinic	Feasibility study, New York	34.2% prediabetes, 6.6% diabetes; 64.6% followed up with PCP
ADA Diabetes Risk Test + PoC HbA1c	University dental clinic (n = 13,519)	35% at risk; 34.9% prediabetes, 5.3% T2DM among those tested
aMMP-8 PoC mouthrinse + questionnaire	Periodontology clinic, Greece (n = 69)	AUC improved from 0.683 to 0.759 for prediabetes detection
FINDRISC + capillary FBG referral model	Private dental clinics, Malaysia (n = 142)	30.3% new prediabetes and 21.2% T2DM among referral completers
DentDi interprofessional model (dental + primary care)	Seven sites, Stockholm, Sweden (n = 372)	27% elevated glucose; 89 prediabetes, 12 T2DM identified
Clinical risk model (periodontitis stage + BMI + family history + smoking)	Tertiary dental clinic, Singapore (n = 1,074)	AUC 0.717; advanced periodontitis as independent predictor

## Conclusions

Prediabetes produces a recognizable and clinically accessible constellation of oral signs, including periodontal attachment loss, salivary dysfunction, and dysbiosis of the oral microbiome. The evidence reviewed here establishes that each of these manifestations is detectable through routine dental examinations or straightforward chairside adjunctive tests that are already available in most dental practices. The dose-dependent relationship between periodontitis staging and HbA1c elevation, the availability of aMMP-8 PoC testing that simultaneously diagnoses periodontal risk and flags glycemic dysregulation, and the demonstrated diagnostic yield of interprofessional referral models all converge to support the dental office as a first-line venue for opportunistic prediabetes identification in high-risk patients. Early detection enables timely lifestyle interventions that can delay or prevent the onset of T2DM and its complications. Dental professionals are uniquely positioned to bridge the substantial gap between undiagnosed metabolic diseases and timely diagnosis. The field as a whole stands to benefit from integrating systematic glycemic screening into routine periodontal assessment protocols.
